# Application of surface plasmon resonance imaging to monitoring G protein-coupled receptor signaling and its modulation in a heterologous expression system

**DOI:** 10.1186/s12896-016-0266-9

**Published:** 2016-04-12

**Authors:** Yuki Nonobe, Tomoki Yokoyama, Yuji Kamikubo, Sho Yoshida, Nozomi Hisajima, Hiroaki Shinohara, Yuki Shiraishi, Takashi Sakurai, Toshihide Tabata

**Affiliations:** Laboratory for Medical Information Sensing, Graduate School of Science and Engineering, University of Toyama, 3190 Gokufu, Toyama, Toyama 930-8555 Japan; Department of Pharmacology, Juntendo University School of Medicine, Hongo 2-1-1, Bunkyo-ku, Tokyo 113-8421 Japan; Laboratory for Bioelectronics, Graduate School of Innovative Life Science, University of Toyama, 3190 Gokufu, Toyama, Toyama 930-8555 Japan

**Keywords:** G protein-coupled receptor, Glutamate, Adenosine, Surface plasmon resonance imaging, Heterologous expression, Neuron, Synaptic plasticity

## Abstract

**Background:**

G protein-coupled receptors (GPCRs) are ubiquitous surface proteins mediating various biological responses and thus, important targets for therapeutic drugs. GPCRs individually produce their own signaling as well as modulate the signaling of other GPCRs. Real-time observation of GPCR signaling and modulation in living cells is key to molecular study of biological responses and pharmaceutical development. However, fluorescence imaging, the technique widely used for this purpose, requires a fluorescent dye which may inhibit biological responses or a fluorescent-tagged target protein created through time-consuming genetic manipulation. In this study, we applied two-dimensional surface plasmon resonance (SPR) imaging to monitoring the translocation of protein kinase C (PKC), a major GPCR-coupled signaling molecule in the widely used HEK293 cell lines and examined whether the signaling of, and, modulation between heterologously expressed GPCRs can be measured without fluorescent labeling.

**Results:**

We cultured HEK293 cells on the gold-plated slide glass and evoked SPR at the interface between the cell’s plasma membrane and the gold surface with incident light. The translocation of activated native PKC to the plasma membrane is expected to alter the incident angle-SPR extent relation, and this could be detected as a change in the intensity of light reflection from the specimen illuminated at a fixed incident angle. Direct activation of PKC with 12-O-tetradecanoylphorbol-13-acetate increased the reflection intensity. This increase indeed reported PKC translocation because it was reduced by a pre-treatment with bisindolylmaleimide-1, a PKC inhibitor. We further applied this technique to a stable HEK293 cell line heterologously expressing the GPCRs type-1 metabotropic glutamate receptor (mGluR1) and adenosine A1 receptor (A1R). (*RS*)-3,5-dihydroxyphenylglycine, a mGluR1 agonist, increased the reflection intensity, and the PKC inhibitor reduced this increase. A pre-treatment with (*R*)-*N*^6^-phenylisopropyladenosine, an A1R-selective agonist suppressed mGluR1-mediated reflection increase. These results suggest that our technique can detect PKC translocation initiated by ligand binding to mGluR1 and its modulation by A1R.

**Conclusions:**

SPR imaging turned out to be utilizable for monitoring GPCR-mediated PKC translocation and its modulation by a different GPCR in a heterologous expression system. This technique provides a powerful yet easy-to-use tool for molecular study of biological responses and pharmaceutical development.

## Background

G protein-coupled receptors (GPCRs) constitute a large protein superfamily with more than 1000 candidate genes in the human genome [1]. GPCRs mediate various biological responses and thus, are important targets for therapeutic drugs. Most GPCRs can produce intracellular signaling as monomers or homomeric oligomers. Moreover, many GPCRs form heteromeric complexes with different types of GPCRs and produce intriguing signaling through inter-GPCR modulation [2, 3]. Real-time observation of GPCR signaling and inter-GPCR modulation in living cells is key to molecular study of biological responses and pharmaceutical development (e.g., ref. [4]). However, fluorescence imaging, the technique widely used for this purpose, requires a fluorescent dye which may inhibit biological responses (e.g., ref. [5]) or a fluorescent-tagged target protein created through time-consuming genetic manipulation (e.g., ref. [6]).

**Fig. 1:**
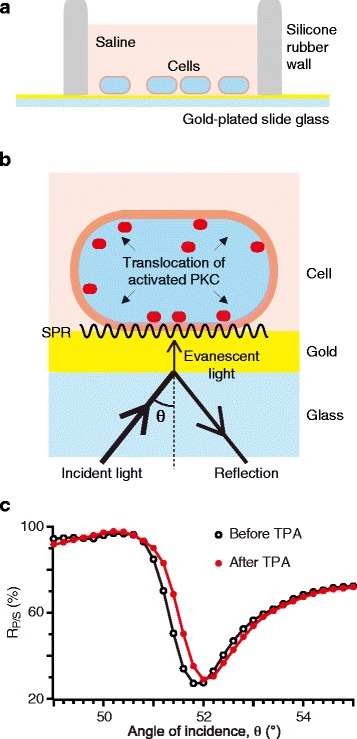
Experimental set-up. **a** HEK293 cells were cultured on a 50 nm-thick gold-plated slide glass. The cell chamber was surrounded with a silicone rubber wall attached to the slide glass. **b** Configuration of SPR imaging. SPR takes place, consuming the energy of the evanescent light derived from the P-polarized component of the incident light. PKC accumulated on the plasma membrane alters the mode of SPR, and this results in changes in the consumption of the evanescent light energy and the intensity of the reflection of the P-polarized light. Theta, angle of incidence. **c** Sample plots of *R*_*P/S*_ against the angle of incidence (average of 5 ROIs) before and after a 15-min application of the potent PKC activator TPA (100 nM)

Two-dimensional surface plasmon resonance (SPR) imaging may provide a ready-to-use tool to explore GPCR signaling and modulation in intact cells. Since pioneered by Liedberg et al., SPR imaging has been utilized for cell-free immunoassay to detect a specific protein in a sample solution [7, 8]. Typically, a sample solution is examined on a gold-plated slide glass with an antibody on its surface. Incident light given to the slide glass evokes the resonant oscillation of conduction electrons at the interface between the sample solution and the gold surface (SPR) (cf. Fig. 1b). When the target protein is trapped by the antibody on the gold surface, the relation of the angle of incidence to SPR extent (so-called SPR curve) will alter. With a fixed angle of incidence, such an alteration results in a decrease or increase of the consumption of the incident light energy for evoking SPR and an increase or decrease of the light reflection from the specimens, respectively. Thus, the light reflection intensity can be taken as a measure of the amount of the trapped protein. Recently, Hide’s group [9] and Shinohara’s group [10] revealed that the accumulation of intracellular proteins near the plasma membrane of the cells cultured on a gold-plated slide glass can also affect the SPR curve (Fig. 1a, b). The latter group further used SPR imaging to detect translocation of native protein kinase C (PKC) in response to 12-O-tetradecanoylphorbol-13-acetate (TPA), a PKC agonist, or an agonist for native PKC-coupled GPCR in PC12 cells [10, 11]. When activated, conventional-type PKC is known to translocate from the cytosol to the plasma membrane (e.g., ref. [12]) and the resultant accumulation of PKC near the plasma membrane is thought to increase the reflection [11]. PKC translocation can be taken as a measure of PKC activity. Besides, PKC translocation itself is an important step of biological responses because the target proteins of PKC-mediated phosphorylation such as ionotropic glutamate receptor reside on the plasma membrane (see below).

If PKC translocation monitoring with SPR imaging is applicable to heterologous expression systems, it will be of great benefit to biological researchers and drug developers. In such a system, one can analyze the functions of specific GPCR signaling cascades of interest in a reduced cellular environment free from other signaling cascades. In this study, we explored this possibility in the widely used heterologous expression system based on human embryo kidney-derived HEK293 cell lines. We used type-1 metabotropic glutamate receptor (mGluR1) and adenosine A1 receptor (A1R) as a model of interplaying GPCRs [13, 14]. In mammalian cerebellar Purkinje neurons, mGluR1 receives the excitatory neurotransmitter glutamate and activates PKC via the G_q/11_ protein-phospholipase C cascade. This signaling triggers long-term depression of Purkinje cell’s postsynaptic glutamate-sensitivity (glu-LTD), a cellular basis for cerebellar motor learning [15]. It is shown that mGluR1 forms complexes with A1R [13, 14, 16], which receives adenosine in the cerebrospinal fluid [17, 18] and activates G_i/o_ protein. We have revealed that A1R activation leads to suppression of glu-LTD [14]. Such inter-GPCR modulation would be important because it may serve as the regulatory mechanism of learning behavior. Although circumstantial evidence suggests that ligand-stimulated A1R attenuates mGluR1 intracellular signaling [14, 19], there was no direct demonstration of the effect of A1R activation on the activity of mGluR1-coupled signaling molecule. We assessed the ability of SPR imaging to detect PKC translocation initiated by ligand binding to mGluR1 as well as A1R-mediated modulation of mGluR1-mediated PKC translocation.

## Results and discussion

### Experimental set-up

HEK293 cell lines could be maintained on the uncoated gold-plated glass (Fig. 1a,b). Heterologous expression of the GPCRs could be induced in these cultured cells within the measurement chamber (see Methods). Such simple cell handling saved labor and time.

Among the components of the incident light, only one whose polarization occurs parallel to the plane of incidence (P-polarized light) is consumed to evoke SPR. Thus, the intensity of the reflection of the P-polarized light (*R*_*P*_) is sensitive to changes of the SPR curve due to intracellular protein translocation. On the other hand, the intensity of the reflection of the component whose polarization occurs perpendicular to the plane of incidence (S-polarized light; S is the initial of the German word “senkrecht” standing for perpendicular)(*R*_*S*_) is not sensitive to the changes of the SPR curve. We quantify intracellular protein translocation by the ratio of *R*_*P*_ to *R*_*S*_ (*R*_*P/S*_) (see Methods for detail).

We measured *R*_*P/S*_, varying the angle of incidence before and after an application of TPA, a potent PKC activator (100 nM) (Fig. 1c). In the following analyses, the angle of incidence was fixed at the value with which TPA-induced change in *R*_*P/S*_ became maximal. In this study, we once polished the imager’s prism to maintain the image quality. The angles of incidence for the maximal *R*_*P/S*_ change were 51.4 ° and 50.3 ° before and after the polishing, respectively.

### Detection of PKC translocation

**Fig. 2:**
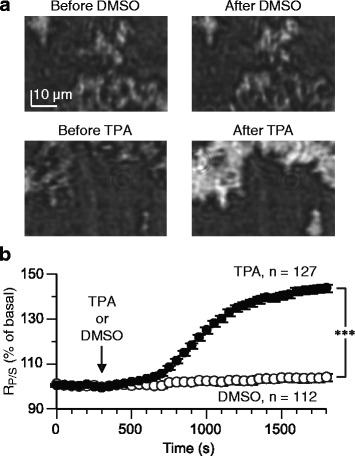
PKC activation results in an increase in *R*_*P/S*_. (**a** and **b**) TPA (100 nM) but not the control vehicle (DMSO) alone increased *R*_*P/S*_ in non-transfected HEK293T cells. In this and following figures, the test agent-containging saline was dropped into the measurement chamber after a 5-min basal measurement; the test agent continued to be present in the chamber onward. **a** Sample images of the reflection of the P-polarized light before and after a 25-min application of the labeled agent. **b** Mean time-plots of *R*_*P/S*_. Arrow, onset of test agent application. Error bars, ±SEM. ***, *p* < 0.0001 at 25 min of the test agent application onset (van der Waerden rank sum test)

In non-transfected HEK293T cells, a 25-min application of the control vehicle {dimethyl sulfoxide (DMSO)} alone little altered *R*_*P/S*_ at the application onset and onward for 25 min. The change of *R*_*P/S*_ at the end of the 25-min DMSO application was only +3.8 ± 1.1 % (mean ± SEM; *n* = 112) of the basal level expressed by the average over a 5-min period prior to the DMSO application (Fig. 2a, b). The raw basal *R*_*P/S*_ was 57.6 ± 0.8 % and the raw *R*_*P/S*_ at the end of the 25-min DMSO application 59.4 ± 0.8 %. In different non-transfected cells of the same batches of cultures, a 25-min application of TPA (100 nM) increased *R*_*P/S*_ by +43.6 ± 1.7 % (*n* = 127) (Fig. 2a, b). The raw basal *R*_*P/S*_ was 55.0 ± 0.8 % and the raw *R*_*P/S*_ at the end of the 25-min TPA application 77.7 ± 0.7 %. The extent of *R*_*P/S*_ change at the end of the 25-min application was significantly different between the control vehicle- and TPA-applied cells (*p* < 0.0001, Van der Waerden rank sum test). The polarity of the *R*_*P/S*_ change (i.e., increase) after PKC activation was consistent with the previous report [11].

**Fig. 3:**
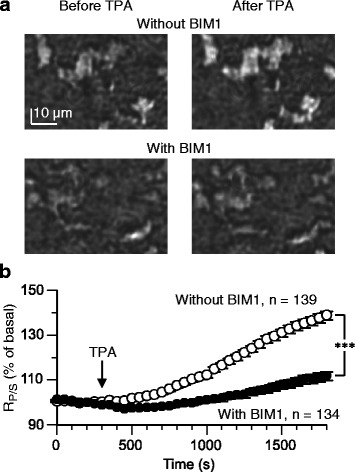
*R*_*P/S*_ increase is due to PKC translocation. **a** and **b** The effect of TPA (100 nM) was examined in non-transfected HEK293T cells pretreated with saline containing the PKC inhibitor BIM1 (300 nM) or the control vehicle alone. BIM1 continued to be present in the saline throughout the measurement. The data were obtained from cultures different from those in Fig. 2. **a** Sample images of the reflection of the P-polarized light. **b** Mean time-plots of *R*_*P/S*_. Error bars, ±SEM. ***, *p* < 0.0001 at 25 min of the TPA application onset (van der Waerden rank sum test)

In non-transfected cells of different batches of cultures, a 25-min application of TPA (100 nM) increased *R*_*P/S*_ by +38.3 ± 2.1 %, (*n* = 139) (Fig. 3a, b). The raw basal *R*_*P/S*_ was 49.9 ± 1.1 % and the raw *R*_*P/S*_ at the end of the 25-min TPA application 67.0 ± 0.9 %. In different cells of the same batches of cultures, a 10-min pre-conditioning with bisindolylmaleimide-1 (BIM1), a PKC inhibitor, reduced the extent of TPA-induced *R*_*P/S*_ increase to +11.7 ± 2.0 % (*n* = 134) (Fig. 3a, b). The raw basal *R*_*P/S*_ was 45.8 ± 0.8 % and the raw *R*_*P/S*_ at the end of the 25-min TPA application 50.1 ± 0.9 %. The extent of *R*_*P/S*_ change at the end of the 25-min TPA application was significantly different between the BIM1-untreated and -treated cells (*p* < 0.0001, Van der Waerden rank sum test). These results suggest that the *R*_*P/S*_ increase indeed reports the translocation of activated PKC. In this study, the cells were not transfected with exogenous PKC gene. Thus, the *R*_*P/S*_ increase was produced by endogenous PKC in HEK293 cells [20].

### Expression of A1R and mGluR1 in HEK293am cells

For analysis of mGluR1- and A1R-mediated responses, we used HEK293am cells, a previously established stable cell line containing the tetracycline-controlled transcriptional activation system and mGluR1 and A1R genes [16]. This cell line was designed to express these GPCRs only when stimulated with a tetracycline derivative such as doxycycline. We used the inducible expression system because cell lines expressing the exogenous GPCRs under the control of constitutive promoters were often lost [16].

**Fig. 4:**
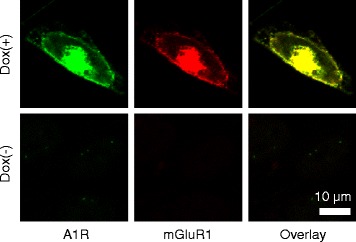
Expression of mGluR1 and A1R in HEK293am cells. Confocal images of doxycycline-treated or -untreated HEK293am cells immunostained with anti-A1R and anti-mGluR1 primary antibodies and fluorescent dye-conjugated secondary antibodies

We confirmed the expression of A1R and mGluR1 in HEK293am cells using immunocytochemistry. HEK293am cells displayed both immunoreactivities for A1R and mGluR1 on their surface when stimulated with doxycycline (Fig. 4, Dox(+)). The absence of immunoreactivities for these GPCRs in doxycycline-untreated cells (Fig. 4, Dox(-)) suggests that this cell line lacks native mGluR1 and A1R. This allowed us to focus on the signaling of heterologously expressed GPCRs. Once expressed in HEK293am cells, A1R and mGluR1 are thought to form heteromeric complexes as reported in the previous co-immunoprecipitation and Föster resonance energy transfer studies [14, 16].

### Detection of mGluR1-mediated PKC translocation

**Fig. 5:**
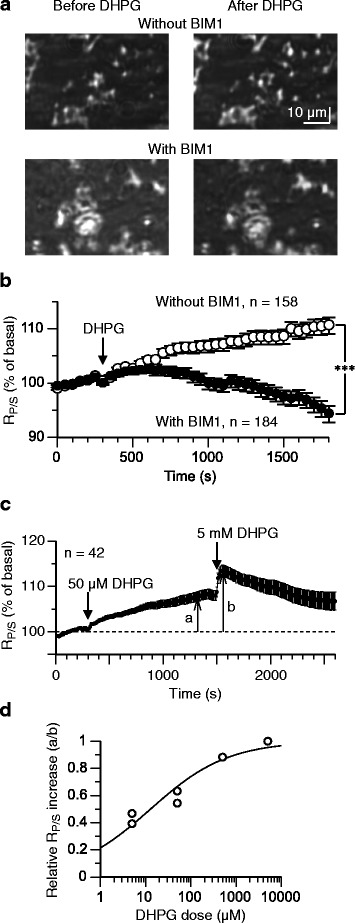
SPR imaging can detect PKC translocation initiated by ligand binding to mGluR1. **a** and **b** In doxycycline-treated HEK293am cells, the mGluR1 agonist DHPG (50 μM) increased *R*_*P/S*_ and this effect of DHPG was abolished with a 10-min pre-conditioning with the PKC inhibitor BIM1 (300 nM). BIM1 continued to be present in the saline throughout the measurement. **a** Sample images of the reflection of the P-polarized light. **b** Mean time-plots of *R*_*P/S*_. Error bars, ±SEM. ***, *p* < 0.0001 at 25 min of the DHPG application onset (van der Waerden rank sum test). **c** and **d** Dose-dependence of DHPG-induced *R*_*P/S*_ increase. **c** Sample mean response of HEK293am cells to serial applications of a test dose (50 μM) and a reference dose (5 mM) of DHPG. On such a mean time-plot, the extents of quasi-maximal *R*_*P/S*_ increases with the test and reference doses (*a* and *b*, respectively) were measured. **d** Dose-response relation depicted using the ratio *a/b*. Each dot indicates the data obtained from the mean response of 17-42 ROIs. Sigmoid curve, Hill function fitted to the data

In doxycycline-treated HEK293am cells, a 25-min application of (*RS*)-3,5-dihydroxyphenylglycine (DHPG), a group-I mGluR agonist, increased *R*_*P/S*_ by +10.6 ± 2.2 % (*n* = 158) (Fig. 5a, b). The raw basal *R*_*P/S*_ was 55.7 ± 1.1 % and the raw *R*_*P/S*_ at the end of the 25-min DHPG application 60.3 ± 1.3 %. This DHPG-induced *R*_*P/S*_ increase was not an artifact because the saline containing the control vehicle (water) alone did not increase *R*_*P/S*_ in doxycycline-treated HEK293am cells (*n* = 23, a different batch of culture; raw basal *R*_*P/S*_, 54.5 ± 1.5 %; raw *R*_*P/S*_ at the end of the 25-min application, 53.2 ± 1.6 %; data not illustrated). In different doxycycline-treated HEK293am cells of the same batches of cultures, a 10-min pre-conditioning with the PKC inhibitor BIM1 abolished the effect of DHPG (-5.7 ± 1.5 %, *n* = 184) (Fig. 5a, b). The raw basal *R*_*P/S*_ was 56.3 ± 0.9 % and the raw *R*_*P/S*_ at the end of the 25-min DHPG application 52.9 ± 1.2 %. The extent of *R*_*P/S*_ change at the end of the 25-min DHPG application was significantly different between the BIM1-untreated and -treated cells (*p* < 0.0001, Van der Waerden rank sum test). These results suggest that SPR imaging can detect PKC translocation initiated by ligand binding to mGluR1. In addition, in the presence of BIM1, *R*_*P/S*_ decreased below the basal level (raw value, 56.3 ± 0.9 %) at the late phase of the measurement (56.1 ± 1.1 to 52.9 ± 1.2 % at 15–25 min of the DHPG application onset). A possibility is that BIM1 not only hampered DHPG-induced PKC activation but also attenuated the basal activity of PKC as previously shown in HEK293 cells (e.g., ref. [21]).

In doxycycline-treated HEK293am cells of different batches of culture, the relative extent of DHPG-induced *R*_*P/S*_ increase changed in a DHPG-dose dependent manner (Fig. 5c, d), suggesting that SPR imaging can be used for quantitative evaluation of GPCR signaling. The microscopic dissociation constant and Hill coefficient estimated from the Hill equation fitted to the dose-response data were 12.8 μM and 0.51, respectively (Fig. 5d). It is noteworthy that desensitization was evident with the highest dose (5 mM) of DHPG (Fig. 5c). This might be due partly to the kinetic property of mGluR1 because desensitization is observed commonly for mGluR1-mediated cation channel current and intracellular Ca^2+^ mobilization induced by high doses of DHPG in cerebellar neurons [22].

**Fig. 6:**
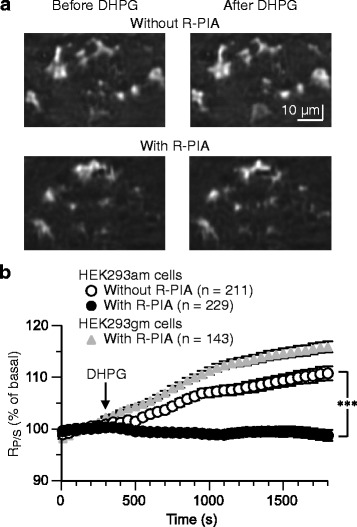
A1R activation leads to suppression of mGluR1-mediated PKC activation. **a** and **b** In doxycycline-treated HEK293am cells, a 25-min application of DHPG (50 μM) increased *R*_*P/S*_. A 10-min pre-conditioning with the A1R antagonist R-PIA (500 nM) abolished the DHPG (50 μM)-induced *R*_*P/S*_ increase. R-PIA continued to be present in the saline throughout the measurement. The data were obtained from cultures different from those in Fig. 5. **a** Sample images of the reflection of the P-polarized light. **b** Mean time-plots of *R*_*P/S*_. Error bars, ±SEM. ***, *p* < 0.0001 at 25 min of the DHPG application onset (van der Waerden rank sum test). Plot with gray triangles, the response of the HEK293gm cells treated with doxycycline and pre-conditioned with R-PIA (5 μM)

It is reported that doxycycline may inhibit multiple intracellular signaling pathways [23]. Thus, replacement with the doxyclycline-containing culture medium with the doxycycline-free saline before the measurement could possibly relieve the cells from such doxycycline-induced inhibition during the measurement. This alone or together with DHPG stimulation could produce a pseudo response. However, non-transfected HEK293T cells treated with doxycycline (2 μg/ml, 32 h) prior to the measurement did not display a *R*_*P/S*_ increase in response to DHPG (50 μM) (*n* = 25; raw basal *R*_*P/S*_, 53.7 ± 1.1 %; raw *R*_*P/S*_ at the end of the 25-min DHPG application, 52.8 ± 1.2 %; data not illustrated). This result suggests that the DHPG-induced *R*_*P/S*_ increases observed in the HEK293am cells (Figs. 5 and 6) were indeed mGluR1-mediated responses but not the pseudo responses.

### Detection of modulation of mGluR1-mediated PKC translocation by A1R

In doxycycline-treated HEK293am cells of different batches of cultures, a 25-min application of DHPG again increased *R*_*P/S*_ by +10.7 ± 1.4 % (*n* = 211) (Fig. 6a,b). The raw basal *R*_*P/S*_ was 58.9 ± 0.8 % and the raw *R*_*P/S*_ at the end of the 25-min DHPG application 64.5 ± 1.0 %. In different doxycycline-treated cells of the same batches of cultures, a 10-min pre-conditioning with (*R*)-*N*^6^-phenylisopropyladenosine (R-PIA), an A1R-selective agonist, abolished DHPG-induced *R*_*P/S*_ increase (-1.3 ± 1.1 %, *n* = 229) (Fig. 6a, b). The raw basal *R*_*P/S*_ was 57.0 ± 0.8 % and the raw *R*_*P/S*_ at the end of the 25-min DHPG application 56.5 ± 1.0 %. The extent of *R*_*P/S*_ change at the end of the 25-min DHPG application was significantly different between the R-PIA-untreated and -treated cells (*p* < 0.0001, Van der Waerden rank sum test). This result suggests that A1R activation leads to suppression of mGluR1-PKC signaling.

PKC is an essential mediator for induction of cerebellar LTD. PKC-mediated phosphorylation is thought to trigger endocytosis of ionotropic glutamate receptor, the final stage of cererellar LTD induction [15]. Thus, the previously reported negative effect of A1R agonist on glu-LTD [14] can be ascribed at least in part to suppression of mGluR1-mediated PKC activation by A1R (Fig. 6). Moreover, we observed this suppression in the non-neuronal cells, suggesting that A1R-mGluR1 functional interplay may occur independently of the neuron-specific cellular environment.

We confirmed that A1R indeed mediated the effect of R-PIA, using a HEK293 cell line that expressed mGluR1 and G_q/11_ protein-coupled gastrin-releasing peptide receptor (GRPR) but not A1R (HEK293gm cells). Although pre-conditioned with a high dose (5 μM) of R-PIA for 10 min, doxycycline-treated HEK293gm cells displayed a *R*_*P/S*_ increase in response to DHPG (50 μM). The extent of this increase (at the end of the 25-min DHPG application, +15.9 ± 1.0 %, *n* = 143) was comparable with that of the HEK293am cells (Fig. 6b). The raw basal *R*_*P/S*_ was 70.3 ± 0.9 % and the raw *R*_*P/S*_ at the end of the 25-min DHPG agent application 80.4 ± 0.7 %.

### Reproducibility and stability of measurement

The extent of TPA-induced *R*_*P/S*_ increase was similar between the different batches of cultures (~50 % at 25 min of the application onset, Figs. 2b vs. 3b). As well, the extent of DHPG-induced *R*_*P/S*_ increase was similar between the different batches of cultures (for 50 μM, ~10 % at 25 min of the application onset, Figs. 5b vs. 6b). These results indicate the high reproducibility of the measurement. The control vehicle did not change *R*_*P/S*_ at the application onset and onward for 25 min (Fig. 2b), indicating that the measurement is robust against physical shock due to test drug application and can stably monitor the response of living cells for a long time. With the easiness-to-use and reliability, SPR imaging in heterologous expression cell preparations may provide a useful experimental paradigm for investigation of GPCR signaling.

## Conclusions

In HEK293 cell lines, SPR imaging could detect PKC translocation resulting from direct activation of PKC itself or initiated by ligand binding to heterologously expressed mGluR1. SPR imaging could also detect the modulation of heterologously expressed mGluR1-mediated PKC translocation by heterologously expressed A1R. SPR imaging in heterologous expression cells provides a powerful yet easy-to-use tool for analyzing GPCR-coupled intracellular signaling and inter-GPCR modulation in living cells.

## Methods

### Cell preparation

HEK293 cell lines were passaged every 3 or 4 days. The cells were detached from the old dish (diameter, 60 mm; 353002, Falcon, Becton, Dickinson and Company, Franklin Lakes, NJ, USA) by sequential treatments with ethylenediaminetetraacetic acid (0.22 w/v %)–containing phosphate-buffered saline (PBS) and a trypsin solution (TrypLE Express, 12605-010, Life Technologies). The detached cells were suspended in a Dulbecco’s modified Eagle medium (08458-45, Nacalai Tesque, Kyoto, Japan) containing 10 v/v % fetal bovine serum (FBS)(12483-012, Life Technologies), 100 units/ml penicillin G potassium (26239-42, Nacalai Tesque), and 100 μg/ml streptomycin sulfate (32204-92, Nacalai Tesque). The cell suspension was poured into a new dish containing the same medium, and the cells were maintained at a temperature of 37 °C, a CO_2_ concentration of 5 %, and a humidity of 96 %.

For analysis of mGluR1- and A1R-mediated responses, we used HEK293am cells, a previously established stable cell line containing the tetracycline-controlled transcriptional activation system and mGluR1 and A1R genes [16]. This cell line expressed these receptors only when stimulated with a tetracycline derivative such as doxycycline. We used the inducible expression system because cell lines expressing the exogenous GPCRs under the control of constitutive promoters were often lost [16]. For selection of HEK293am cells, the medium was supplemented with 15 μg/ml blasticidin S hydrochloride (KK-400, Kaken Pharmaceutical, Tokyo, Japan), 120 μg/ml hygromycin B (09287-84, Nacalai Tesque), and 480 μg/ml G418 (09380-44, Nacalai Tesque). For negative control, we used HEK293gm cells, a newly created stable cell line containing the tetracycline-controlled transcriptional activation system and mGluR1 and GRPR genes. We maintained the HEK293gm cells as in the case of the HEK293am cells.

For SPR imaging, 500 μl of the cell suspension was prepared as above and placed in a chamber that was constructed with a silicone rubber wall (volume, 0.88 ml; 032039, flexi PERM, Greiner Bio-One, Kremsmünster, Austria) attached to a 50 nm-thick gold-plated slide glass (012190, BAS, Tokyo, Japan) 2 days prior to the measurement and cultured until the measurement. To induce gene expression in HEK293am or HEK293gm cells, the old medium was replaced with the fresh medium supplemented with 2 μg/ml doxycycline (324385, Calbiochem, La Jolla, CA, USA) 1 day prior to the measurement. The individual cells were used for only one measurement session. To minimize possible influence of passage-to-passage or batch-to-batch deviation in cell responsiveness, the data were compared within cultures of the same passage generation of the same batch.

### Immunocytochemistry

HEK293am cells cultured in a glass-based dish were treated with doxycycline for 16 h as above. The cells were fixed at 4 °C for 30 min with 0.1 M sodium phosphate buffer containing 4 w/v % paraformaldehyde. The fixed cells were rinsed with PBS and then incubated at room temperature for 30 min in PBS containing 0.1 v/v % Triton X-100 and 5 v/v % FBS. The cells were then probed at 4 °C overnight with 1 μg/ml primary antibodies against mGluR1 and A1R (mouse anti-mGluR1 antibody, 610965, BD Bioscience, San Jose, CA, USA; in-house rabbit antibody against amino acids 309–329 of rat A1R [14]), and subsequently stained at room temperature for 2.5 h with 5 μg/ml fluorescent dye-conjugated secondary antibodies (Alexa Fluors 448 and 594, Molecular Probes, Eugene, OR, USA). Cell images were captured using a confocal laser microscope (SP5/TCS, Leica Microsystems, Wetzlar, Germany).

### SPR imaging

We performed two-dimensional SPR imaging according to refs. [10, 11]. Briefly, the cell chamber was rinsed three times with saline consisting of (in mM): 147 NaCl, 3 KCl, 2 CaCl_2_, 1 MgCl_2_, 10 HEPES, and 10 D-glucose (pH adjusted to 7.4 with NaOH) and then filled with 270 μl of the saline. The chamber was placed on a SPR imager (2D-SPR04B, NTT Advanced Technology, Kawasaki, Kanagawa, Japan) with matching oil (refractive index, 1.8000; 12787, Cargille Laboratories, Cedar Grove, NJ, USA). Imaging was performed at a room temperature of ~25 °C. The bottom of the chamber was illuminated with 770-nm incident light (light source output current, 50 mA) at a fixed angle of incidence. The image of the reflection from the specimen was captured using the charge-coupled device camera built in the imager (objective lens magnification, x7; cooling temperature, 0 °C). Each image frame had a size of 639 x 479 pixels and a gray scale of 12 bits and was stored in bmp format.

We sequentially captured the images of *R*_*P*_ with an exposure time per frame of 1 or 5 s and an interval of 10 or 50 s (test session). For reference, we captured the images of *R*_*S*_ immediately before and after the test session (reference sessions).

### Drug preparation and application

TPA (4174S, Cell Signaling Technology, Danver, MA, USA) was dissolved in DMSO (13407-45, Nacalai Tesque) to a concentration of 2 μM and kept at -50 °C until use. BIM1 (203291, Calbiochem) was dissolved with water to a concentration of 300 μM and kept at 4 °C until use. DHPG (0342, Tocris Cookson, Bristol, UK) was dissolved in water to a concentration of 5 mM and kept at -50 °C until use. R-PIA (P4532, Sigma-Aldrich, St. Louis, MI, USA) was dissolved into water to a concentration of 500 μM and kept at 4 °C until use. On the day of experiment, the stock solution of the test drug was diluted into the saline to a concentration 10 times higher than the final concentration. An application was done by dropping 30 μl of the drug-containing saline to the cell chamber.

### Data analysis

The reflection intensity was analyzed using ImageJ 64 bit software (National Institute of Health, Bethesda, MD, USA) or the dedicated analyzing software supplied by the manufacturer of the SPR imager. A group of tightly gathered cells was selected as a single region of interest (ROI). A ROI was an ellipse typically with 50–200 pixels (~3–12 μm^2^). Mean *R*_*P*_ over each ROI was calculated for each image frame of the test session. Mean *R*_*S*_ over the ROI was calculated for each image frame of the reference sessions and averaged over these sessions. To evaluate the extent of change, *R*_*P*_ was normalized to the averaged *R*_*S*_ for each ROI (raw *R*_*P/S*_) and then further %-normalized, taking the mean over a 5-min period prior to the test agent application as 100 % for each ROI.

To measure the dose-response relation of the DHPG-induced *R*_*P/S*_ increase, we first applied a test dose (5–500 μM) of DHPG and then applied the reference dose (5 mM) of DHPG to the same cells. For each ROI, the mean extent of the quasi-maximal *R*_*P/S*_ increase with a test dose was expressed relative to that with the reference dose. In the cases of the test doses, the extent was measured at 17 min of the DHPG application onset. In the case of the reference dose, the extent was measured at the peak of response because there was evident desensitization. A Hill equation in a form of $R=\frac{{\left[L\right]}^{n_H}}{{\left({K}_D\right)}^{n_H}+{\left[L\right]}^{n_H}}$, where *R*, *L*, *K*_*D*_, and *n*_*H*_, were the relative extent of response, DHPG dose, microscopic dissociation constant, and Hill coefficient, respectively, was fitted to the data plotted in Fig. 5d using Excel 2008 software (Microsoft, Redmond, WA, USA) with Solver plug-in.

Each group of numerical data is expressed as mean ± SEM. A difference between a pair of the data groups was examined with a non-parametric statistical test (van der Waerden rank sum test) using JMP 9 software (SAS, Cary, NC, USA) because the original distribution of the data might be distorted through %-normalization.

### Ethics approval and consent to participate

Not applicable.

### Consent for publication

Not applicable.

## Availability of data and material

All the data obtained in this study are presented in the main body of this article and there are no supporting data available outside the main body of this article.

## Abbreviations

A1R: adenosine A1 receptor
BIM1: bisindolylmaleimide-1
DHPG: (*RS*)-3,5-dihydroxyphenylglycine
DMSO: dimethyl sulfoxide
FBS: fetal bovine serum
GPCR: G protein-coupled receptor
GRPR: gastrin-releasing peptide receptor
HEK293am cell: stable human embryo kidney 293 cell line that expresses mGluR1 and A1R under the control of the tetracycline-controlled transcriptional activation system
HEK293gm cell: stable human embryo kidney 293 cell line that expresses mGluR1 and GRPR under the control of the tetracycline-controlled transcriptional activation system
mGluR1: type-1 metabotropic glutamate receptor
PBS: phosphate-buffered saline
PKC: protein kinase C
P-polarized light: light whose polarization occurs parallel to the plane of incident
ROI: region of interest
*R*_*P*_: intensity of the reflection of the P-polarized light
R-PIA: (*R*)-*N*^6^-phenylisopropyladenosine
*R*_*P/S*_: relative *R*_*P*_ normalized to *R*_*S*_
*R*_*S*_: intensity of the reflection of the S-polarized light
S-polarized light: light whose polarization occurs perpendicular to the plane of incident
SPR: surface plasmon resonance
TPA: 12-O-tetradecanoylphorbol-13-acetate
